# Finite Element Simulations of Hard-On-Soft Hip Joint Prosthesis Accounting for Dynamic Loads Calculated from a Musculoskeletal Model during Walking

**DOI:** 10.3390/ma11040574

**Published:** 2018-04-09

**Authors:** Alessandro Ruggiero, Massimiliano Merola, Saverio Affatato

**Affiliations:** 1 Department of Industrial Engineering, University of Salerno, Via Giovanni Paolo II, nr. 132, 84084 Fisciano, Italy; 2 Medical Technology Laboratory, IRCCS—Rizzoli Orthopaedic Institute, Via di Barbiano, 1/10, 40136 Bologna, Italy; massimiliano.merola@ior.it (M.M.); affatato@tecno.ior.it (S.A.)

**Keywords:** total hip arthroplasty, musculoskeletal multibody model, dynamic loading, finite element analysis, radial clearance, dry and wet friction

## Abstract

The hip joint replacement is one of the most successful orthopedic surgical procedures although it involves challenges to overcome. The patient group undergoing total hip arthroplasty now includes younger and more active patients who require a broad range of motion and a longer service lifetime for the replacement joint. It is well known that wear tests have a long duration and they are very expensive, thus studying the effects of geometry, loading, or alignment perturbations may be performed by Finite Element Analysis. The aim of the study was to evaluate total deformation and stress intensity on ultra-high molecular weight polyethylene liner coupled with hard material head during one step. Moving toward in-silico wear assessment of implants, in the presented simulations we used a musculoskeletal multibody model of a human body giving the loading and relative kinematic of the investigated tribo-system during the gait. The analysis compared two frictional conditions -dry and wet and two geometrical cases- with and without radial clearance. The loads and rotations followed the variability of the gait cycle as well as stress/strain acting in the UHWMPE cup. The obtained results allowed collection of the complete stress/strain description of the polyethylene cup during the gait and calculation of the maximum contact pressure on the lateral edge of the insert. The tensional state resulted in being more influenced by the geometrical conditions in terms of radial clearance than by the variation of the friction coefficients due to lubrication phenomena.

## 1. Introduction

Total Hip Replacement (THR) is the most successful application of biomaterials in the short term in order to alleviate pain, restore joints, and increase functional mobility in diseased traumatized articulations [1,2,3]. A major limiting factor to the service life of THRs remains the wear of the polyethylene acetabular cup [4,5]. Preclinical endurance testing has become a standard procedure to predict the mechanical performance of new devices during their development. Wear tests are performed on different materials and designs used in prosthetic implants [1,6,7,8] to obtain quality assessment and acquire further knowledge about the tribological processes of joint prostheses. The objective of these investigations is to find out the wear rate and its dependence on the test conditions. In order to obtain realistic results, a wear test should reproduce the in vivo working conditions on the artificial implants [9].

It is well known that wear tests that are close to the in vivo conditions have a long duration and elevate costs [1,10,11]. The wear simulation is run for several million cycles, considering that one million cycles corresponds to one year in vivo [12,13,14]. The running-in period encompasses approximately the first half a million cycles; the steady-state wear is assessed by measuring the wear after the running-in period [15]. Wear is evaluated either gravimetrically, determining the weight loss of the components, or by measuring the volume of the material that has been removed, e.g., wear pit dimensions [9,16]. Lubricant absorption or creep of the loaded components, especially for polyethylene, is considered. Wear measurements are normally done at lubricant change stops, e.g., every 500,000 cycles.

Finite Element Analysis (FEA) has been widely used in many areas of biomechanics and medical engineering [17]. The numerical modelling tool of finite element analysis has been widely applied to study the behavior of articular cartilage, joints, and bone structures under compressive and tensile stresses. Structural applications include the design and development of joint prosthesis and fracture fixation devices. FEA enables to investigate parameters and boundary conditions, which are not accessible experimentally nor analytically. It has been applied to orthopedic devices to gain a deep understanding of the behavior of the bone-implant system and to support the design and pre-clinical testing of new devices. Thus, computational wear simulation can be a valuable complement to wear tests, e.g., in predictions of the lifetime of prostheses evaluating the effects of geometry, loading, or alignment perturbations.

As the first attempt to apply FEA to the orthopedic field dates back to 1972 [18], there have been four decades of developing and improving this methodology, along with the increase of computational power. Nevertheless, to make in silico wear simulation meaningful, better wear models are needed through advanced study design and corroboration with in vitro testing. The most intricate aspect of such models is the loading conditions acting on the implants, as their knowledge is still not fully reached. Few hip replacements have been subjected to direct load measures from Bergmann et al. [19,20,21], which limits the result validity to the subjects of the study. A more general load analysis can be obtained by muscular skeletal models, as it has been done by the authors [22], to study knee implants, or by van der Ploeg et al. [23], who used the multibody results as input for a FEA studying the micro-motions of a femoral stem.

In this work, a musculoskeletal multibody model was used in order to estimate the loads acting on the hip joint during walking. A finite elements analysis was then conducted using these loads and kinematic inputs. The study reports the results in terms of total deformation and stress intensity of an acetabular polyethylene liner coupled with a femoral head of a hard (ideally rigid) material.

## 2. Materials and Methods

### 2.1. Gait Cycles and Loads

In this study, the authors used a musculoskeletal modelling software to estimate loads acting on the hip joint during level walking. The simulation was performed with AnyBody Modelling System™ (AMS) [24]. To calculate the joint forces, knowledge of kinematic data and ground reaction forces that are used as input is required. These data are collected in gait analysis laboratories using special motion capture tools [25], which allows the measurement of a subject’s gait through cameras that monitor markers on the subject’s skin.

In inverse dynamics, the motion and the external loads on the body are known, and the aim is determining the internal forces. However, not enough equilibrium equations are available to find all the unknowns of the problem, therefore the calculation of the muscle forces is possible by the so-called redundancy problem. The solution of the muscle recruitment problem in the inverse dynamics approach is generally formulated as an optimization problem of the form:

(1) $$\min G\left(f^{\left(M\right)}\right)$$

with

(2) Cf=d

(3) $$0\leq f_{i}^{\left(M\right)}\leq N_{i},i\;\in \;\left\{1,\;\ldots ,\;n^{\left(M\right)}\right\},$$

where *G* is the objective function (1), i.e., the assumed criterion of the recruitment strategy of the central nervous system, stated in terms of the muscle forces, and minimized with respect to all unknown forces in the problem, $f={\left(\;f^{\left(M\right)T}\;f^{\left(R\right)T}\right)}^{T}$, (i.e., muscle forces and joint reactions). Equation (2) is the dynamic equilibrium equations, which enter as constraints into the optimization. *C* is the coefficient matrix for the unknown forces/moments in the system, *d* is a vector of the known applied loads and inertia forces. The non-negativity constraints on the muscle forces, Equation (3), states that muscle can only pull, not push and the upper bounds limit their capability, so *N*_1_ is the strength of the muscle.

The most popular forms of the objective function G, calculated from the relative intensity and normalized for each muscle, are the polynomial criteria and the soft saturation criteria per Siemienski et al. [26]:

(4) $$G\left(f^{\left(M\right)}\right)=\;\overset{n^{\left(M\right)}}{\underset{i=1}{\sum }}{\left(\frac{f_{i}^{\left(M\right)}}{N_{i}}\right)}^{p}$$

with p=2, since it is established in the literature as predicting reasonable muscle activation patterns for the type of analyzed trial.

All segments of the biomechanical system are modelled as rigid bodies, neglecting effects such as the wobbly masses of soft tissues.

### 2.2. Finite Element Modelling

**Figure 1:**
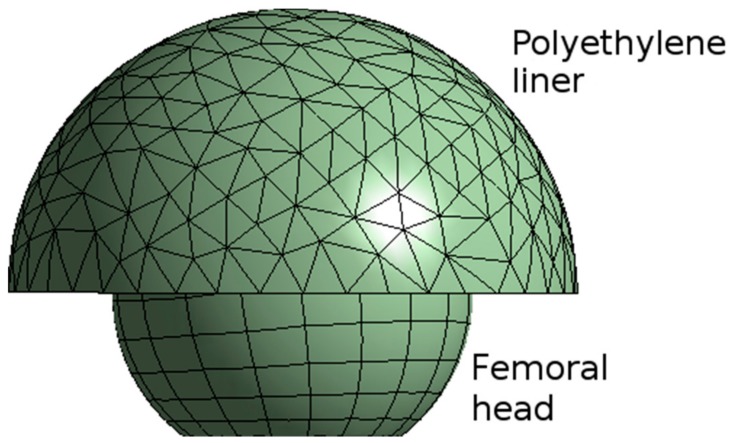
Mesh model of the femoral head and the polyethylene liner.

The finite elements model was realized through Ansys^®^ Workbench commercial software (v.18.1, ANSYS Inc., Canonsburg, PA, USA). To minimize the computational complexity there were considered only the two major bearing components in a hip implant, namely the femoral head and the acetabular cup. The presence of the pelvic bone has been neglected, since its influence has a least effect on contact pressure [27]. The bodies of the FE model are shown in Figure 1, the mesh was realized through quadratic tetrahedral elements. Mesh convergence tests were performed, resulting in a total number of elements and nodes of 2012 and 3283, respectively, having 383 contact elements.

To solve the contact problem the Augmented Lagrange algorithm was used, considering an asymmetric behavior, which is recommended for the solution of frictional contacts. The femoral head was modelled as a rigid body, whereas for the polyethylene insert of the cup it was selected the Ultra-High-Molecular-Weight-Polyethylene (UHMWPE) GUR 1050 material, as it is one of the main polymer materials used for implant applications [28,29]; Table 1 summarizes its main parameters.

The material is assumed to be homogenous and isotropic. Even if roughness plays a key role on the tribological behavior of hip implants [30,31], the model surfaces are considered smooth, as in most of the models found in literature, for the ease of solution.

The study was conducted to understand the stress behaviour in dry conditions, as the presence of lubricant modifies the pressure distribution on the surfaces. However, as further knowledge, the influence of the friction coefficient was introudced, comparing a dry and a lubricated regime, namely, a DRY case, i.e., no lubrication, and a WET case, i.e., lubricated conditions. The mean values of the friction coefficient in lubricated conditions were extracted from experimental studies on the prosthesis tribological pairs [32,33]. Thus, a dry friction value of 0.13 and a mean wet friction value of 0.05 was selected.

**Figure 2:**
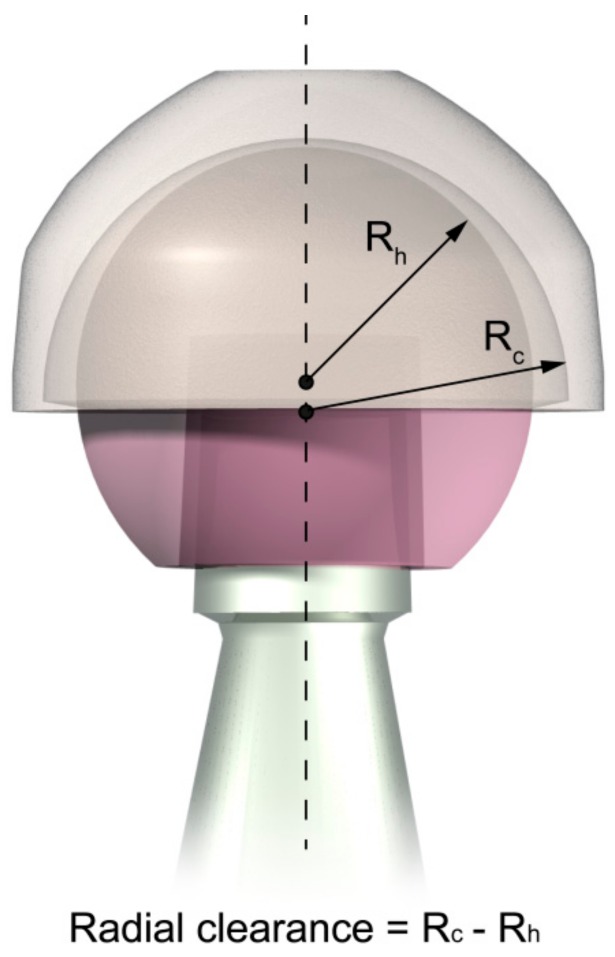
Radial clearance is the difference in radius of the acetabular cup and the femoral head. In this image the clearance is amplified for a better understanding.

The femoral head had a diameter of 28 mm, whereas the acetabular cup had a thickness of 5 mm. Moreover, two geometrical configurations of the coupled bodies were studied, and the simulations were executed considering the presence of the radial clearance (CC condition) and not considering it (NC condition). Radial clearance is the difference between the radius of the acetabular cup and the one of the femoral head (see Figure 2). Radial clearance, when considered, was 0.5 mm [34]; the results of the two configurations were then compared.

**Figure 3:**
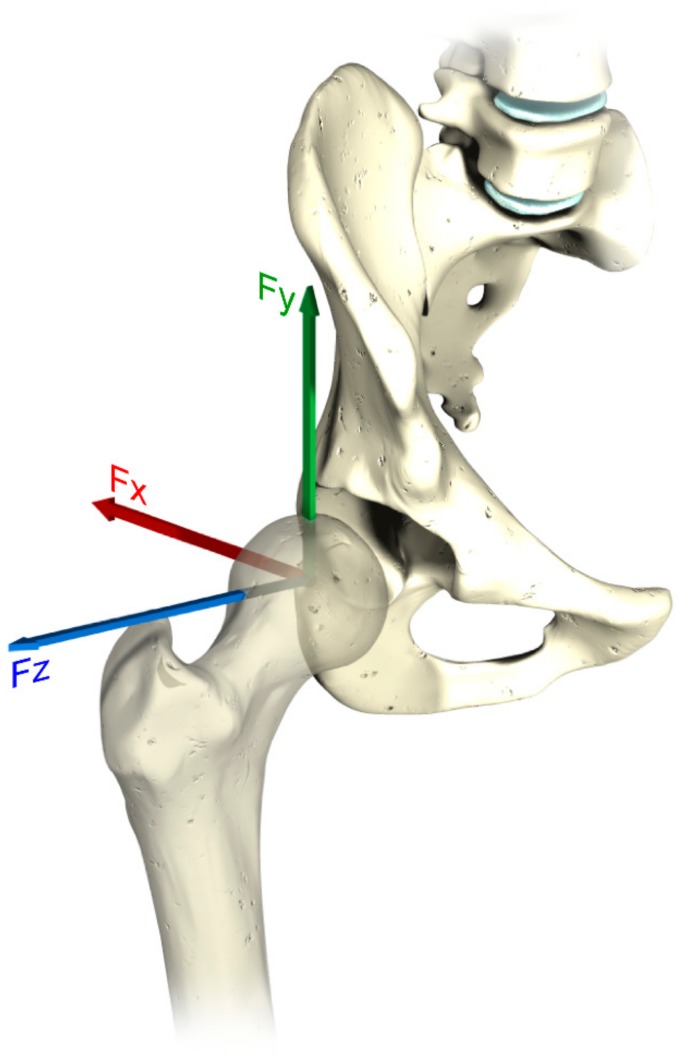
The three force vectors represented on a femoral head.

The input data obtained by the multibody system were evaluated in a local coordinates system, which follows the movements of the femoral head. As the FE model requires a global coordinates system, a conversion was performed considering well-known geometric transformations [35]. Further force components and mesh orientations are defined with respect to a pelvic reference frame that coincides with the true anatomic superior, anterior, and lateral directions (see Figure 3). The tensional state and the total deformation were evaluated on the inner surface of the polyethylene liner.

## 3. Results

The forces and the rotations taking place along the three degrees of freedom were gained from the multibody analysis. The force components are the ones depicted in Figure 3. The three rotations around the axes are the *Flexion*/*Extension* (around *z* axes), *Abduction*/*Adduction* (around *x* axes) and *Inward*/*Outward* (around *y* axes).

**Figure 4:**
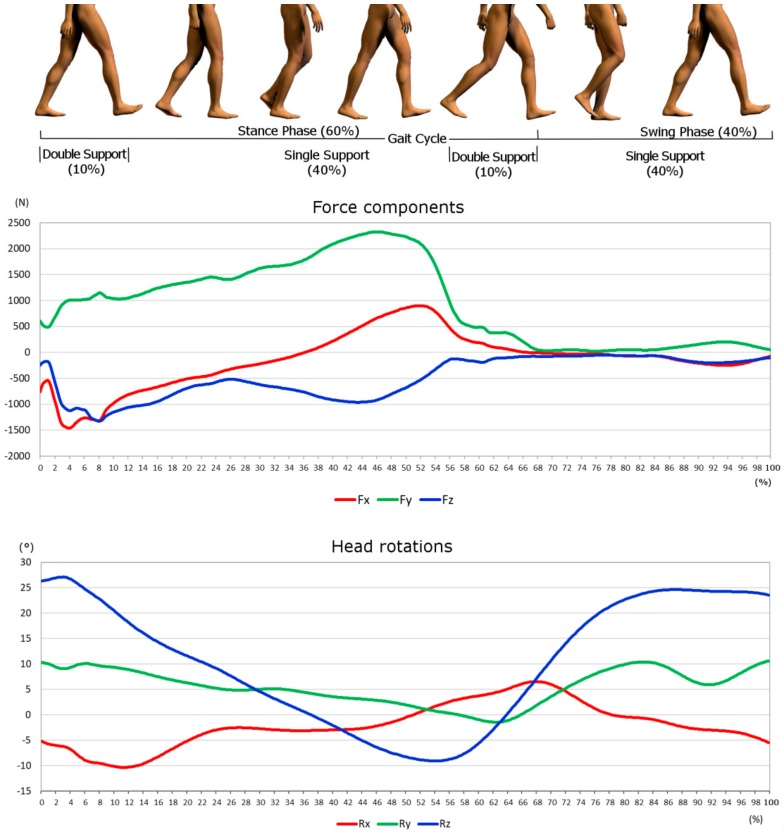
Forces components, in red Fx, in green Fy, in blue Fz. Head rotations, around *x* axes in red, *y* axes green and *z* axes blue.

In Figure 4 the force components and the rotations derived from the model are shown. It is noticeable that the highest rotation is the *Flexion*/*Extension*, whereas the highest load is along the *y* axes. Forces and rotations so obtained were used as dynamic inputs for the finite element model.

**Figure 5:**
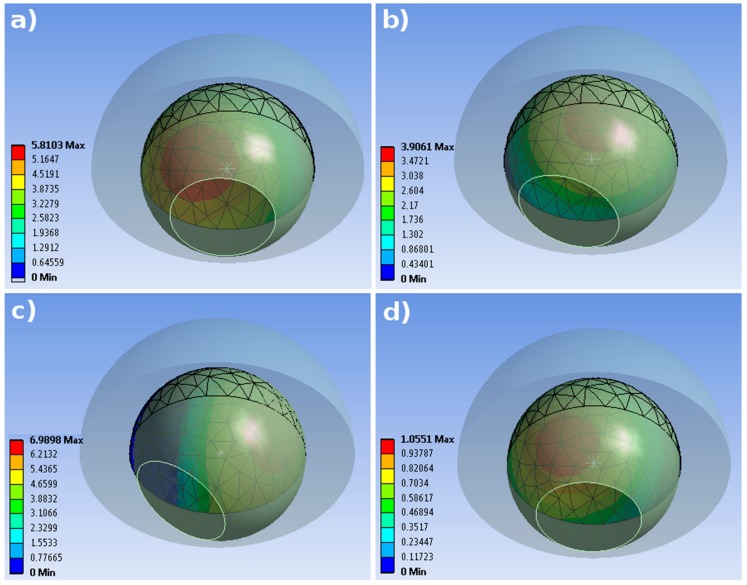
Pressure distribution on the internal surface of the acetabula cup. (**a**) NC case, dry condition; (**b**) NC case, wet condition; (**c**) CC case, dry condition; (**d**) CC case, wet condition.

In Figure 5 the pressure distribution on the internal surface of the cup in different instants of the cycle is shown, with regards to the dry NC condition as exemplificative case. Along with the different orientation of the femoral head it is possible to observe the pressure distribution on the polyethylene liner. Its highest values are found at 8% and 48% of the cycle (respectively Figure 5a,c). In the latter case the highest level of the *Anterior*/*Posterior* force was also found (see Figure 4), and the pressure is more concentrated in the edge zone of the insert. In the other two images, Figure 5b,d, at 26% and 93% of the cycle, the pressure reaches lower values and its mostly located in the central part of the inner hemisphere. The other geometrical and frictional cases are here omitted for brevity, but they presented a similar distribution of pressure, only leading different intensity of the tensional state.

**Figure 6:**
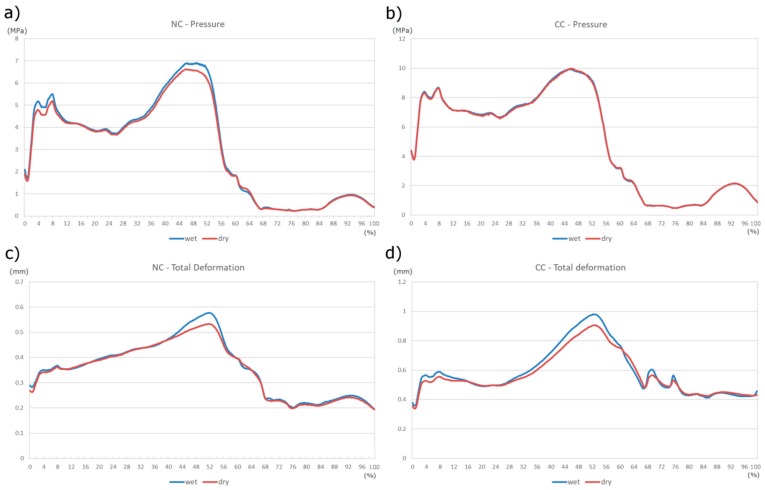
Comparison of result in the NC (**a**,**c**) and CC (**b**,**d**) cases, between the dry and wet conditions.

**Figure 7:**
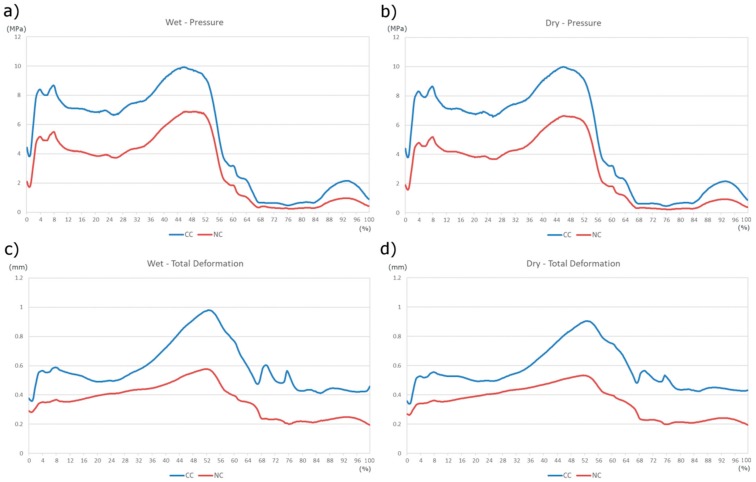
Comparison of result in the wet (**a**,**b**) and dry (**c**,**d**) condition, between the CC and NC cases.

Figure 6 and Figure 7 present a summary of the maximum values of pressure and total deformation, comparing the two friction conditions and the two geometric configurations. In Figure 6a the maximum value of pressure is shown, for each part of the cycle. The maximum value throughout the walking cycle is equal to 7 MPa and it is found around 48% of the way through the cycle—agreeing with the dynamic analysis. A slight difference was found between the two friction cases, showing a higher peak value in the wet condition. In Figure 6c the maximum value of the total deformation is displayed; its highest value is again related to the wet case, reaching almost 0.6 mm. In Figure 6b,d the comparisons are shown, in terms of pressure and deformation, between the two friction cases considering the presence of the radial clearance (CC). In Figure 6b, the comparison highlights the almost complete lack of difference in the two friction conditions; the highest value reached is almost 10 MPa. However, in Figure 6d the curves have some differences, showing slightly higher values of total deformation in wet condition (maximum value of 1 mm and 0.9 mm for the wet and the dry case, respectively).

In Figure 7 the comparison of the two geometrical conditions is presented, with and without radial clearance (CC and NC, respectively). In Figure 7a, considering the boundary lubrication, the maximum pressure plot shows the large divergence in the two geometrical solutions. The highest values are almost 10 MPa and 6.7 MPa, for the CC and NC respectively. As well as the curves in Figure 7c, where the total deformation is shown, the highest values are found for the CC condition where it reaches almost 1 mm, whereas NC gives back at the most 0.6 mm. The curves in Figure 7b also brings out the divergences in the two geometrical configurations under dry friction, the pressure in CC being higher than the one found in NC; the values are almost the same as those already described in the wet condition. Figure 7d reports the maximum values of the total deformation, clearly showing the difference in the CC and NC conditions.

## 4. Discussion

In this work, the forces and rotations acting on the hip joint during a complete gait cycle were first evaluated by solving the inverse dynamic problem. These data agree with the walking cycle variability [25], showing how the joint results more loaded during the stance phase than in the swing phase, when there is almost no contact between the foot and the ground. Furthermore, the highest loads is the axial (along the *y* axes) as expected and confirmed by a comparison with the standard ISO 14242-1:2012 [36]. Afterwards, these loads and rotation were used as dynamical input in the finite element model, obtaining the pressure distribution and the elastic deformation on each node of the surface during the whole gait cycle. The stress state of the internal surface of the UHMWPE liner reaches peaks of the order of 10 MPa, being in accordance with literature results [37,38,39]. Several authors [36,40,41] used simplified hip kinematic and gait load, such as one-dimensional vertical load or considered only the flexion/extension rotation [40], which does not represent actual physiological loading, plus these studies rely on ISO standards at the expense of a flexible design. In this regard, the original contribution of the study is the acquisition of stress and deformation distribution based on a multibody model. This offers the possibility to apply a wide variety of gaits and to consider the peculiarity of a specific class of patients, e.g., older or younger people. This could lead to the design of prosthesis which account the requirement of a patient, by simply characterize his activity through the multibody and finite elements model.

The comparison of the different working conditions allowed the conclusion that the presence of radial clearance strongly influences the tensional state of the coupled surfaces. Whereas, for a complete understanding of the lubrication influence on the tribology of hip implants the Reynolds equations must be involved [42], as varying the coefficient of friction does not allow the establishment of a sensible difference in results. In literature, the contact is usually assumed to be frictionless, justifying this hypothesis with negligible variation in contact pressure distribution. However, the presence of a frictional force has an influence to the location of the nominal contact point, as demonstrated by Mattei and Di Puccio in [35].

The reasons for the differences across the two geometrical conditions are ascribable to the different tensional state of the surfaces. In the geometry without radial clearance a conformal contact is realized, meaning that the contact is distributed along an area. On the other hand, when the radial clearance is considered, the contact become non-conformal, which implies that the contact is limited to a small area that increases its extension as the deformation rises. These differences in the area size led to the distinction of the pressure values, shown in the previous paragraph. As stated by Teoh et al. [37] the radial clearance plays a vital role in the wear process, finding that its extreme values (0.001 and 0.5 mm) led to the highest level of wear. In the study by Tudor et al. [43] the difference between the conditions of small loads with high clearances is highlighted, where the contact pressure tends to a Hertzian distribution, and high loads with small clearances, where the contact pressure tends to have a hyperbolic distribution. Furthermore, concerning the influence of the frictional force, in the study by Teoh et al. [37] they found that varying the friction coefficient between 0 and 0.3, only yielded a slightly variance of the wear rate.

## 5. Conclusions

This work is meant as a first step forward to the simplified hip kinematic and gait load. In this work we presented a dynamic load, derived by a multibody technique applied to a musculoskeletal model, considering the variability of the load direction during gait movement.

The main conclusions of the study are:

- multibody technique applied to a musculoskeletal model was proven to be a valid instrument to obtain a flexible design of implant, leading to the evaluation of load for a specific demanding task;
- load components and rotations match the variability between the stance and the swing phase of the leg during the gait;
- highest values of pressure on the inner surface of the polyethylene insert are found near its edge side;
- tensional state and elastic deformation are mainly influenced by the radial clearance rather than the friction coefficient.

The results of the present work offer the possibility to extend, in further studies, the range of kinematics and dynamics; an additional improvement could include lubrication to apply this set-up in a realistic scenario.

## Figures and Tables

**Table 1:** UHMWPE GUR 1050 attributes.

Density	Young’s Modulus	Poisson’s Ratio	Bulk Modulus	Shear Modulus	Tensile Yield Strength	Tensile Ultimate Strength
(kg·m^−3^)	(MPa)	(-)	(MPa)	(MPa)	(MPa)	(MPa)
930	690	0.43	1640	241	21	40

## Nomenclature

THR	Total Hip Replacement
AMS	AnyBody Modelling System
FEA	Finite Element Analysis
UHMWPE	Ultra-High-Molecular-Polyethylene
*G*	objective function
*f^(M)^*	muscle forces
*f^(R)^*	joint reaction
*d*	vector of applied loads and inertia forces
*N_1_*	strength of the muscle
